# The Efficacy of Regeneration Oil and Almond Oil on Split-Thickness Skin Graft Donor Sites: A Single-Blinded Randomized Controlled Trial

**DOI:** 10.3390/clinpract13030059

**Published:** 2023-05-25

**Authors:** Karoline Riedler, Andrzej Hecker, Birgit Bauer, Christa Tax, Daniel Georg Gmainer, Anna-Lisa Pignet, Lars-Peter Kamolz, David Benjamin Lumenta

**Affiliations:** 1 University Hospital Graz, 8036 Graz, Austria; karo.riedler@gmx.at (K.R.);; 2 Division of Plastic, Aesthetic and Reconstructive Surgery, Department of Surgery, Medical University of Graz, 8036 Graz, Austria; 3 Research Unit for Digital Surgery, Division of Plastic, Aesthetic and Reconstructive Surgery, Department of Surgery, Medical University of Graz, 8036 Graz, Austria

**Keywords:** aromatherapy, essential oil, scar quality, skin care, split-thickness skin graft donor site

## Abstract

Background and Objectives: Essential oils are a complementary treatment and can play an important role in scar care. The aim of this study was to evaluate and compare the efficacy of a new essential oil (regeneration oil) with a control group on scar quality in healed split-thickness skin graft donor sites. Materials and Methods: A single-center blinded randomized controlled study was performed on 30 patients with healed split-thickness skin graft donor site. The patients were randomly allocated into blended regeneration oil (*n* = 14) and pure almond oil (*n* = 16) groups. Application of the assigned oil occurred twice a day for 6 months. Scarring (Patient and Observer Scar Assessment Scale), itching (ITCH Assessment Scale) and scar discoloration (colorimetry) of the donor sites were assessed after 1, 3 and 6 months. Results: We found no statistically significant differences between the groups in any applied parameter. We observed comparable outcomes (scar quality, itchiness, colorit) in healed split-thickness skin graft donor sites for both oils. Conclusions: Regeneration oil and control oil presented comparable results regarding scar quality, itchiness and colorit in healed split-thickness skin graft donor sites after 6 months of application. Both oils are suitable for skin/scar care in split-thickness skin graft donor sites.

## 1. Introduction

Aromatherapy has been used throughout the centuries, and essential oils were a purported method for protection from infectious diseases. Until the middle of the nineteenth century, most of the remedies for chronic diseases, pain and contagious diseases were obtained from plants which were believed to have a healing effect not only on the body but also on the soul. Throughout the centuries, natural healing has been regarded as a part of conventional medicine [1]. Deriving from aromatherapy, essential oils comprise the use of 100% natural essential oils, fatty plant oils, hydrolates and derived care products. The term aromatherapy covers all types of treatment carried out with 100% natural essential oils or fatty plant oils in humans [2,3,4]. While no effects of aromatherapy were found in comparison to standard therapy for dementia, anxiety, hypertension and radiation therapy patients [5,6], a positive effect on well-being in rheumatologic patients was shown [7]. In 2012, Lee, in an overview of systematic reviews, investigated the effect of aromatherapy as an alternative therapy approach. Again, insufficient evidence for the use of aromatherapy and care with essential oils was found [5]. As a standard of skin care after (completed) wound healing and to support the development of skin suppleness, treatment with a fatty ointment or oil is recommended, whereas in regular skin care (no wounds or scars), pure oils are recommended [8].

The aim of this study was to evaluate the efficacy of a new essential oil (regeneration oil) as a topical agent on scar quality in healed split-thickness skin graft (STSG) donor sites in patients over the course of 6 months as assessed by subjective (wound/itch/scar assessment of patient and observers) and objective parameters (colorimetry).

## 2. Materials and Methods

### 2.1. Trial Design and Participants

This was a single-blinded, randomized controlled trial (RCT) conducted at the Division of Plastic, Aesthetic and Reconstructive Surgery, Department of Surgery, Medical University of Graz. The study design and the protocol were approved by the ethical committee of the Medical University of Graz (27-109 ex 14/15). Informed consent was obtained from the study participants prior to the study start. This study is registered in “Deutschen Register Klinischer Studien” (ID: DRKS00030072).

For this study, we finally recruited 30 volunteers (*n* = 30) after completion of wound healing (re-epithelization phase) around 10–14 days postoperatively, who previously had surgery involving STSG on the thigh between March 2015 and July 2016. We included male and female in-patients between 18 and 85 years of age, who had a standardized donor site on the anterior aspect of both thigh and a negative skin irritation test for the used oils.

The exclusion criteria included known allergic reactions to one of the oils used in the study, heart disease affecting cardiac output, active neoplastic disease, congenital and acquired disease of the immune system, anemia (hemoglobin < 10 g/dL or hematocrit < 35%), autoimmune disease, acute/chronic renal failure with serum creatinine levels above 176 mmol/L, renal disease requiring dialysis, hepatic cirrhosis, active liver infections/hepatitis, known alcohol or drug abuse, severely underweight and cachectic patients (BMI < 16), untreated thyroid dysfunction, diabetes mellitus, pregnancy, lactation, denied participation in the study, as well as lack of co-operation.

### 2.2. Procedure

The participants were randomly assigned to an intervention group (regeneration oil) or control group (almond oil) to receive the assigned oil as a topical agent for home use, using the Randomizer for Clinical Trials Tool (https://www.randomizer.at/ (accessed on 25 July 2016) developed at the Medical University of Graz. For concealed allocation, both oils were bottled in identical brown glass bottles with the same dripping devices and labeled with the randomization codes. A total of 50 mL of the respective oils were intended for the care of the respective STSG donor site within the 24-week time frame. Another batch of oil was provided to participants upon request.

The patients were instructed by trained, qualified nursing staff in the handling of the oils and to carry out the application of the assigned oil on the STSG donor site twice a day. For safety reasons and to screen for potential intolerances to one of the oils, topical application (approximately 2 cm^2^) on a healthy skin area was carried out and controlled at the end of the outpatient appointment, before providing patients with their respective oils. With the help of a patient diary (with check boxes for each application), compliance was monitored by checking the patient diary and verifying with the patient whether the assigned oil was regularly applied to the affected site as recommended.

Scarring, itching and colorimetry of the re-epithelized STSG donor site were assessed at the beginning of the study (measurement time point 1: MT1) and then after 4 (MT2), 12 (MT3) and 24 weeks (MT4).

### 2.3. Regeneration Oil/Control Oil

The regeneration oil (Aromapflege GmbH, Pflach, Austria) contains special additives like, Helichrysum italicum flower oil, Lavandula angustifolia oil, Cistus ladaniferus oil, Santalum album oil, Styrax benzoin resin oil, Melaleuca viridiflora leaf oil, Hippophae rhamnoides fruit oil, Rosa canina fruit oil, Argania spinosa oil and Hypericum perforatum extract with Olea europaea oil, all of which are said to accelerate the regeneration of the skin.

The comparative control oil is pure almond oil (oleum amygdalae, Gatt-Koller GmbH, Absam, Austria), which is routinely used in patients with re-epithelized split-thickness graft donor site at the Division of Plastic, Aesthetic and Reconstructive Surgery, Department of Surgery, Medical University of Graz.

### 2.4. Outcome Measures

Study-related outpatient clinic appointments after 4, 12 and 24 weeks were booked in conjunction with routine (=non-study-related) outpatient clinic visits to the Division of Plastic, Aesthetic and Reconstructive Surgery. During these visits, the Patient and Observer Scar Assessment Scale (POSAS) [9], the ITCH assessment scale [10] and photographic documentation for colorimetry were assessed.

Each STSG donor site (scar location: right anterior thigh *n* = 15, left anterior thigh *n* = 15) of the patients was evaluated during each follow-up using the POSAS Scale from the perspective of the patient (POSAS_SELF_) as well as the observer (POSAS_OBS_). Changes to the STSG donor site over time can be assessed from the perspective of the patient and observer. The observer scale (POSAS_OBS_) consists of seven discrete items regarding scar characteristics: vascularity, pigmentation, thickness, relief, surface area, pliability, and overall opinion.

The patient scale (POSAS_SELF_) measures symptoms (itch, pain), scar characteristics (color, stiffness, thickness, irregularity), and how the scar compares to normal skin. Both scales are classified from 1 to 10, with 1 representing normal skin and 10 representing very poor scar/skin. The total score of POSAS sums each individual scar characteristic and ranges from 6 (best possible scar) to 70 (worst possible scar) [9].

The ITCH assessment scale [10] was used to assess the degree of itching at each control point. On the basis of this Likert-type scale, the itching is graded from 0 to 4, whereby 0 stands for well-being and no itching and 4 is pronounced by extremely strong itching which caused the patient to find it impossible to sit still or concentrate.

Photographs were taken under artificial light with identical conditions (white wall) by two professional (employed) photographers using the same single-lens reflex camera (Nikon D 300, Tokyo, Japan) and the same settings (200/50, aperture 7.1), indirect flash (broncolour^®^, Wolfratshausen, Germany). A standardized color chart (X-rite, ColourChecker^®^ Passport, MI, Grand Rapids, USA) was concomitantly photographed. Photographs were calibrated for digital image processing using tonal value correction. For an objective assessment of the color changes of wound areas, the relevant image areas were set as free and non-relevant areas were set as transparent.

For the colorimetric evaluation of the wound and (unaffected) normal skin, the observation of the color histogram was used for the color red (=vascularity). To account for differences over time (=pigmentation), we employed differential comparative values, since the pigmentation of the normal skin also changed during the course of a year (for example, stronger tanning of the skin in summer).

In the quantitative evaluation of the photos, the red tone was evaluated by means of a standardized image analysis process after calibration of the photographs (tonal value correction). Sample images from colorimetric evaluation are shown in Figure S1. The resulting values were transferred to a spreadsheet program (Microsoft^®^ Excel 2010, Redmond, WA, USA) table from the image analysis program (Adobe^®^ Photoshop^®^ CS2, version 9.0, Adobe Systems, San Jose, CA, USA).

The POSAS scale and the ITCH assessment scale are originally in English. Both scales were tested for reliability and validity. The POSAS scale is a proven reliable and valid tool for the assessment of scars [9]. They were both translated into German and back-translated into the original language by a native speaker with a medical background to ensure linguistic validity [11]. There was a 95% match. In 14 items and 19 categories, there were two inconsistencies in the translation, which were resolved in a joint consensus. The ITCH assessment scale is a reliable and valid tool for the detection of itching after burns. The English version also was tested to determine its validity and reliability [10].

### 2.5. Statistical Analysis

All data were collected prospectively and entered into a spreadsheet program (Excel^®^, Microsoft^®^ Cooperation, Redmond, WA, USA). Statistical analysis was performed after transfer to IBM^®^ SPSS^®^ (Statistics 23.0, Armonk, North Castle, NY, USA). To analyze differences between groups in the course of self-reporting POSAS-Scale (POSAS_SELF_) and observational POSAS Scale (POSAS_OBS_), analysis of variances for repeated measurements (four time points) were calculated. Additionally, 95% confidence intervals for the mean of each group for each time point were calculated. For values of colorimetry, similar analysis was performed using three time points. For the analysis of the ITCH assessment scale, values were dichotomized to “no itching” (ITCH value = 0) and “itching” (ITCH value ≥ 1). These dichotomized data were analyzed for each time point separately using χ^2^-tests or Fisher’s exact test. Analysis of questionnaire data were performed using an intention-to-treat approach. If at least the first two examinations were available, the last observed value was recorded at missing values for the following examination dates (last observation carried forward). All *p*-values were based on a two-sided test. To analyze changes in skin color, differences between normal skin and scar tissue were applied. For the photo evaluation, the red tone difference was compared over the course of all three recording times for all 18 persons. *p*-values ≤ 0.05 were considered statistically significant.

## 3. Results

**Figure 1:**
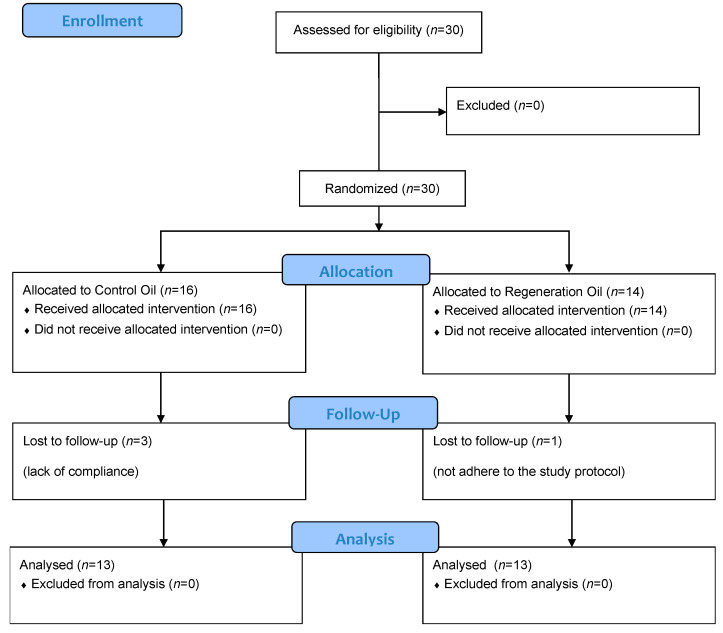
CONSORT flow diagram.

A total of 30 patients were included: 11 women (36.7%) and 19 men (63.3%) of Caucasian origin with a mean age of 50.1 (standard deviation ± 17.9) years. Table 1 shows a descriptive overview of patient comorbidities. We did not observe any allergic reaction or adverse events in response to the applied oils in either group. One participant (regeneration oil) not adhering to the study protocol was not included in the POSAS evaluation (Figure 1). Two participants were lost to follow-up, and one participant was excluded because of non-compliance with the oil application (control oil). The data of three patients were imputed because of missing values. We analyzed photographs using a computer-based colorimetry model in 18 participants (*n* = 10 intervention group (IG), *n* = 8 control group (CG)). The images from excluded cases (*n* = 12) did not meet the criteria required for colorimetric evaluation (e.g., reflections, blurring), and were therefore not included in the colorimetric analyses.

### 3.1. Primary Outcome: POSAS_OBS_

During the course of the study period, there was a significant reduction in POSAS_OBS_ (*p* < 0.001) and within all POSAS_OBS_ sub-categories (vascularity, pigmentation, thickness, relief, pliability, surface area, and overall satisfaction) (*p* < 0.001), indicating a subjective scar improvement according to the observers. The course of the two groups did not differ (F = 78.873, *p* = 0.921; Table 2). Within the sub-categories, there was no statistically significant difference between the two groups (Table S1). There was no statistically significant difference between the two groups (F = 0.560, *p* = 0.462).

### 3.2. Primary Outcome: POSAS_SELF_

During the course of the study period, a statistically significant reduction in the total value (F = 46.486, *p* < 0.001, Table 2) occurred within all POSAS_SELF_ sub-categories (pain, itching, color, stiffness, thickness, irregularity, and overall satisfaction) (*p* < 0.001), indicating a subjective scar improvement observed by the patients. The course of the two groups (intervention group and control group) did not differ (*p* = 0.739) (Table 2). Within the sub-categories, there was no statistically significant difference between the two groups (Table S1). There was no statistically significant difference between the two groups (F = 1.499, *p* = 0.233).

### 3.3. ITCH-Assessment-Scale

A total of 29 persons (MT1: CG *n* = 16, IG *n* = 13) were included in the analysis at measurement time point (MT) 1. 10 of 16 (62.5%) and 9 of 13 (69.2%) participants reported on itching for CG and IG, respectively (*p* = 1.000). For MT2, MT3 and MT4, 27 persons were analyzed (MT2/3/4: CG *n* = 14, IG = 13): at MT 2 itching was experienced in 12/14 (85.7%) and 9/13 (69.2%) persons (*p* = 0.385), at MT3 in 9/14 (64.3%) and 4/13 (30.8%) (*p* = 0.128), for MT 4 3/14 (21.4%) and 2/13 (15.4%) (*p* = 1.000) for CG and IG, respectively (Table 1).

### 3.4. Colorimetry

For the colorimetric evaluation of the scar and (unaffected) normal skin, the observation of the color histogram was used for the color red (=vascularity). A descriptive overview is shown in Table 2. There was a statistically significant change in the vascularity (color red) over time (*p* = 0.010). We found no statistically significant differences in the vascularity (color red) between the two groups (*p* = 0.805) (Table 2).

## 4. Discussion

In this study, we investigated the potential effects of a new essential oil (regeneration oil) and pure almond oil (control oil) in a standardized clinical model (STSG donor site). With a lack of studies using an appropriate methodology to evaluate essential oils, we present, to the best of our knowledge, the first pilot trial demonstrating a statistically significant improvement of patients’ scar self-assessment (POSAS_SELF_, POSAS_OBS_) and itch assessment over time. No significant differences were observed between the oils regarding scar and itch assessment. The color remained constant over the measured time period. While the oils were distinct in terms of smell and appearance (control: odorless and colorless, essential regeneration oil: sweetish-smelling, brown-red), all study participants suspected they were in the group using the essential regeneration oil, eliminating any initial reservations with regard to the blinding of patients.

Interestingly, whilst the subjective assessment of scars demonstrated an improvement over time when either oil was used, no improvement was found by observers (POSAS_OBS_) or by the use of objective support (colorimetry). Most likely, an extension of the measured time period would be required to determine if there were any influences of the oils on color. More importantly, factors dictating the subjective assessment of scars are not primarily based on color, but more so on other factors such as itching or texture [9]. No statistically significant differences were found for itching. While the itch scale is a simple validated tool initially developed for a pediatric setting, its testing in adults is not readily applied [10]. We hypothesize, in view of our findings, that application of either oil resulted equally in an improvement of itching in the study area. Previous research demonstrated that the active utilization of an oil to massage the area resulted in a reduction in itching, taking note of the poor evidence available in the current literature [12].

Our results demonstrated an improvement in the itching with both oils and no method was found to be superior. On the one hand, we cannot exclude that our sample size was too small for detecting any statistically significant differences. In this context, the results of this study can be used to provide data for sample size planning regarding scar care and itching in STSG donor sites after application of regeneration oil or our control oil. On the other hand, there is the possibility that the regeneration oil is not inferior to the control oil. In this case, a study can be planned based on the results to investigate the non-inferiority of the regeneration oil compared to the control oil with a sufficiently planned number of future cases. In a non-inferiority design, the new method is not significantly worse than the reference treatment [13].

Patients benefit from the use of aromatic oils because they are inexpensive, associated health risks are negligible and care with essential oils has been shown to be a needs-oriented and health-promoting care method. Essential oils are derived from natural sources and are readily available, making them a convenient option for skin and scar care [5,6,7,8]. Nevertheless, some individuals may be allergic to certain essential oils, leading to skin irritations or allergic reactions when applied to the skin. Although no single allergic reaction has been reported in this study, it is important to consider this potential side effect before applying essential oils. By means of a simple skin irritation test (topical application of the administering oil on a small area of healthy skin), as carried out in this study, possible allergies can be detected before application. To promote further research on this topic, we provided a study design template in this research area to evaluate differences in a standardized clinical model (STSG donor site) by the use of subjective (scar quality: POSAS_OBS,_ POSAS_SELF_; itching: ITCH assessment scale) and objective (scar discoloration: colorimetry) methods. The follow-up period of 6 months was adequate for capturing the commonly experienced subjective aspects, such as itching and texture, during the early stages of scar maturation. However, it is important to note that long-term aspects, specifically changes in skin color, may persist for a duration exceeding 12 months. This aspect should be considered in future studies [14].

## 5. Limitations

As there is little to no evidence-based research on the effect of essential oils on skin graft donor sites, this study contributed to the development of knowledge of wound regeneration, despite the relatively small sample size. The number of possible participants was restricted by the exclusion of comorbidities (for example diabetes, peripheral occlusive diseases); however, this allowed for the selection of a standardized patient collective, eliminating confounding factors with a negative influence on wound healing. Furthermore, the study contained more male participants and therefore an analysis of differences between the genders was not possible. The observed follow-up was suitable for detecting the most commonly perceived subjective features (itching and texture) of the early scar maturation process; however, long-term aspects (skin color) last for a period of over 12 months [14]. Therefore, future studies should contain at least a 12-month period to possibly detect differences between the analyzed groups. Our presented data can be a valuable source for performing sample size calculations for future studies.

Finally, the essential regeneration oil contained only 5% essential oil additives, in comparison to the higher concentrated control oil. Hypothetically, higher concentrations might have produced more meaningful results but also increased the likelihood of allergic reactions. Since the use of the oils is viewed critically by a wide range of medical disciplines, due to the fear of allergic reactions, it is important to point out that no single allergic reaction has been reported in this study.

## 6. Conclusions

Regeneration oil and the control oil presented comparable results regarding scar quality, itchiness and colorit in healed split-thickness skin graft donor sites after 6 months of application. Therefore, both oils are suitable for skin/scar care in split-thickness skin graft donor sites.

## Figures and Tables

**Table 1:** Descriptive overview of patient comorbidities.

Comorbidities		Control Oil (*n* = 16)	Regeneration Oil (*n* = 14)
Cardiovascular	*n* (%)	4 (25%)	4 (28.6%)
Psychiatric	*n* (%)	4 (25%)	1 (7.1%)
Mild kidney disease	*n* (%)	2 (12.5%)	0 (0%)
Endocrinological	*n* (%)	0 (%)	2 (14.2%)
Rheumatological	*n* (%)	2 (12.5%)	4 (28.6%)
Neurological	*n* (%)	0 (%)	2 (14.2%)
Pulmonary	*n* (%)	1 (6.3%)	2 (14.2%)
Neoplastic	*n* (%)	1 (6.3%)	2 (14.2%)
Metabolic	*n* (%)	4 (25%)	4 (28.6%)

**Table 2:** Descriptive overview of scar quality from the perspective of the observer (POSAS_OBS_), the patient (POSAS_SELF_), itching (ITCH) and colorimetry over a 24-week time period. SD: standard deviation.

Outcome	Baseline	4 Weeks	12 Weeks	24 Weeks
POSAS_OBS_ score Mean (SD)				
Control oil	49.4 (±4.2)	27.2 (±2.8)	18.2 (±2.9)	13.6 (±3.1)
Regeneration oil	46.2 (±4.0)	23.9 (±2.4)	17.0 (±2.3)	12.2 (±2.1)
*p*	0.786	0.378	0.826	0.711
POSAS_SELF_ scores Mean (SD)				
Control oil	39.2 (±4.0)	19.7 (±2.7)	14.9 (±2.1)	12.1 (±1.6)
Regeneration oil	34.8 (±4.0)	17.5 (±1.7)	14.2 (±2.0)	8.9 (±1.1)
*p*	0.766	0.489	0.835	0.134
ITCH rate, in %				
Control oil	62.5	85.7	64.3	21.4
Regeneration oil	69.2	69.2	30.8	15.3
*p*	1.000	0.385	0.128	1.000
Colorimetry scores Mean [confidence interval]				
Control oil		−24.4 [−45.9 to −2.9]	−12.0 [−31.2 to 7.2]	−5.0 [−17.8 to 7.8]
Regeneration oil		−24.3 [−42.5 to −6.1]	−13.6 [−27.0 to −0.2]	−5.1 [−16.9 to 6.7]
*p*		0.385	0.128	1.000

## Data Availability

Detailed data supporting the results are available from the authors.

## Supplementary Materials

The following supporting information can be downloaded at: https://www.mdpi.com/article/10.3390/clinpract13030059/s1, Figure S1: Colorimetric evaluation; Table S1: POSAS sub-categories.
