# Supplementary material for: The Efficacy of Regeneration Oil and Almond Oil on Split-Thickness Skin Graft Donor Sites: A Single-Blinded Randomized Controlled Trial
Source: Clin Pract. 2023 May 25;13(3):648–55. doi: 10.3390/clinpract13030059 (PMC10297259; doi:10.3390/clinpract13030059)
Supplement: Supplementary file 1 [file clinpract-13-00059-s001.zip › clinpract-2290663-supplementary.pdf]

Supplementary Files:

The Efficacy of Regeneration Oil and Almond Oil on Split-Thickness Skin Graft Donor Sites: A Single-Blinded Randomized Controlled Trial

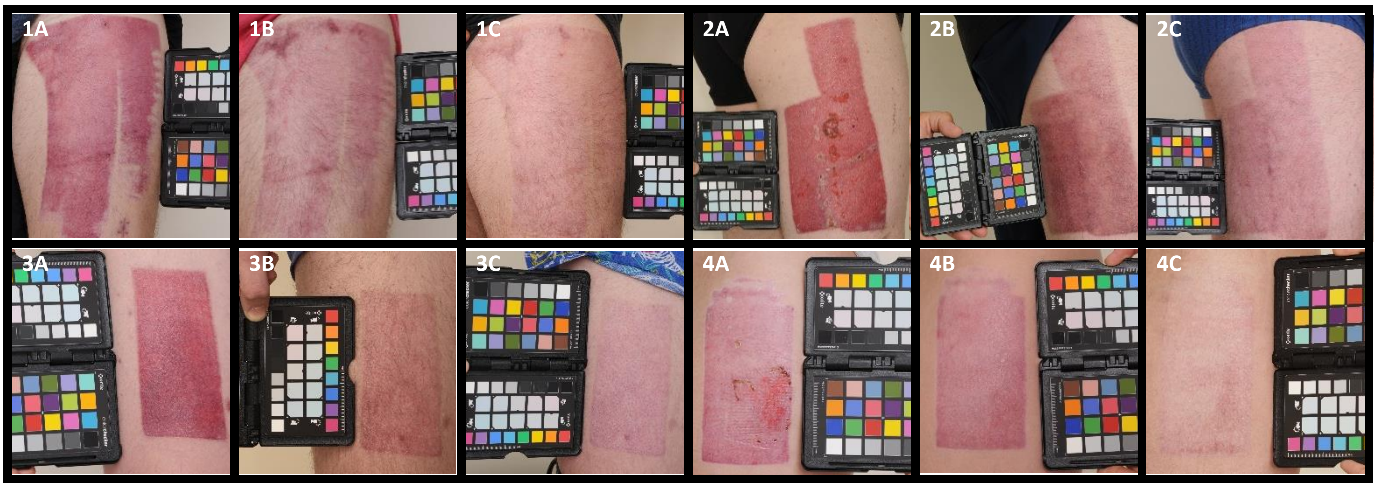

Figure S1. Colorimetric evaluation: sample images from respective follow up appointments (A: 4 weeks B: 12 weeks C: 24 weeks). Control oil group (1 and 2) and regenerative oil group (3 and 4).

	Baseline		4 weeks		12 weeks		24 weeks		p-value
Parameter (mean, SD)	C	R	C	R	C	R	C	R	
POSAS$_{SELF}$ pain	4.7 (+/- 3.5)	3.6 (+/- 2.6)	1.6 (+/- 1.2)	1.6 (+/- 0.9)	1.3 (+/- 0.7)	1.1 (+/- 0.3)	1.0 (0.0)	1.0 (0.0)	0.644
POSAS$_{SELF}$ itching	4.2 (+/- 3.3)	4.3 (+/- 2.7)	2.9 (+/- 1.7)	3.2 (+/- 1.9)	2.3 (+/- 1.7)	1.5 (+/- 0.7)	1.8 (+/- 1.0)	1.1 (+/- 0.3)	0.385
POSAS$_{SELF}$ colour	8.6 (+/- 2.2)	6.5 (+/- 3.0)	6.3 (+/- 2.8)	5.2 (+/- 2.8)	4.7 (+/- 2.6)	4.2 (+/- 2.4)	3.2 (+/- 1.0)	2.3 (+/- 1.0)	0.732
POSAS$_{SELF}$ stiffness	7.1 (+/- 3.3)	6.4 (+/- 3.0)	3.1 (+/- 2.4)	2.6 (+/- 1.2)	1.9 (+/- 1.6)	2.5 (+/- 1.7)	1.6 (+/- 1.0)	1.2 (+/- 0.4)	0.496
POSAS$_{SELF}$ thickness	6.8 (+/- 3.6)	6.4 (+/- 3.2)	2.7 (+/- 2.1)	1.9 (+/- 1.2)	2.1 (+/- 1.6)	2.3 (+/- 1.8)	1.5 (+/- 0.8)	1.2 (+/- 0.4)	0.58
POSAS$_{SELF}$ irregularity	7.0 (+/- 3.4)	6.9 (+/- 2.9)	2.5 (+/- 1.6)	2.75 (+/- 2.2)	1.4 (+/- 0.8)	2.1 (+/- 1.3)	1.7 (+/- 1.1)	1.1 (+/- 0.3)	0.586
POSAS$_{SELF}$ overall satisfaction	7.5 (+/- 2.8)	6.7 (+/- 3.1)	4.3 (+/- 3.1)	4.7 (+/- 1.7)	3.6 (+/- 2.5)	2.8 (+/- 0.9)	2.3 (+/- 1.1)	2.2 (+/- 1.2)	0.658
POSAS$_{OBS}$ vascularity	8.3 (+/- 2.4)	7.1 (+/- 2.9)	3.8 (+/- 1.7)	2.9 (+/- 1.5)	1.6 (+/- 0.8)	1.9 (+/- 1.0)	1.5 (+/- 0.8)	1.3 (+/- 0.5)	0.357

$POSAS_{OBS}$ pigmentation	7.9 (+/- 2.9)	7.7 (+/- 2.5)	4.9 (+/- 1.7)	4.1 (+/- 1.4)	3.6 (+/- 1.1)	2.9 (+/- 1.1)	2.3 (+/- 0.9)	2.4 (+/- 1.1)	0.652
$POSAS_{OBS}$ thickness	8.1 (+/- 2.5)	7.7 (+/- 2.3)	4.1 (+/- 1.4)	3.3 (+/- 1.7)	2.5 (+/- 1.0)	2.4 (+/- 1.0)	1.4 (+/- 0.9)	1.4 (+/- 0.7)	0.729
$POSAS_{OBS}$ relief	8.1 (+/- 2.6)	7.5 (+/- 2.4)	4.5 (+/- 1.7)	4.2 (+/- 1.6)	2.9 (+/- 1.2)	2.7 (+/- 1.3)	2.0 (+/- 0.6)	1.8 (+/- 0.7)	0.904
$POSAS_{OBS}$ pliability	8.1 (+/- 2.5)	7.5 (+/- 2.5)	3.7 (+/- 1.2)	4.2 (+/- 1.9)	2.3 (+/- 1.1)	2.7 (+/- 1.3)	1.7 (+/- 0.7)	1.4 (+/- 0.7)	0.492
$POSAS_{OBS}$ surface area	8.1 (+/- 2.6)	7.5 (+/- 2.4)	4.5 (+/- 1.7)	4.2 (+/- 1.6)	2.9 (+/- 1.2)	2.7 (+/- 1.3)	2.0 (+/- 0.6)	1.8 (+/- 0.7)	0.904
$POSAS_{OBS}$ overall satisfaction	8.3 (+/- 2.4)	7.3 (+/- 2.9)	4.5 (+/- 1.5)	4.3 (+/- 1.3)	3.1 (+/- 1.4)	3.0 (+/- 1.2)	1.9 (+/- 0.7)	1.6 (+/- 0.7)	0.492

Table S1. POSAS sub-categories: descriptive overview of scar quality all POSAS sub-categories from the perspective of the observer ($POSAS_{OBS}$), and the patient ($POSAS_{SELF}$). SD = Standard deviation; C= Control oil; R= Regeneration oil.
